# Association of rotating night shift work, CLOCK, MTNR1A, MTNR1B genes polymorphisms and their interactions with type 2 diabetes among steelworkers: a case–control study

**DOI:** 10.1186/s12864-023-09328-y

**Published:** 2023-05-03

**Authors:** Qinglin Li, Shengkui Zhang, Han Wang, Zhende Wang, Xiaohong Zhang, Yongbin Wang, Juxiang Yuan

**Affiliations:** 1 Department of Epidemiology and Health Statistics, School of Public Health, North China University of Science and Technology, Hebei Tangshan, People’s Republic of China; 2 Tianjin Baodi District Center for Disease Control, Tianjin, People’s Republic of China; 3 Department of Public Health Crisis Management, School of Public Health, Shandong Province, Weifang Medical University, Weifang, People’s Republic of China; 4 Department of Epidemiology and Health Statistics, School of Public Health, Xinxiang Medical University, Henan Province Xinxiang, People’s Republic of China

**Keywords:** Rotating night shift work, Circadian rhythm, Melatonin receptors, Polymorphisms, Interaction

## Abstract

**Background:**

The purpose of this study is to investigate the association of rotating night shift work, CLOCK, MTNR1A, MTNR1B genes polymorphisms and their interactions with type 2 diabetes among steelworkers.

**Methods:**

A case–control study was conducted in the Tangsteel company in Tangshan, China. The sample sizes of the case group and control group were 251 and 451, respectively. The logistic regression, log-linear model and generalized multifactor dimensionality (GMDR) method were used to investigate the interaction between circadian clock gene, melatonin receptor genes and rotating night shift work on type 2 diabetes among steelworkers. Relative excess risk due to interaction (RERI) and attributable proportions (AP) were used to evaluate additive interactions.

**Results:**

Rotating night shift work, current shift status, duration of night shifts, and average frequency of night shifts were associated with an increased risk of type 2 diabetes after adjustment for confounders. Rs1387153 variants in MTNR1B was found to be associated with an increased risk of type 2 diabetes, which was not found between MTNR1A gene rs2119882 locus, CLOCK gene rs1801260 locus and the risk of type 2 diabetes. The association between rotating night shift work and risk of type 2 diabetes appeared to be modified by MTNR1B gene rs1387153 locus (RERI = 0.98, (95% CI, 0.40–1.55); AP = 0.60, (95% CI, 0.07–1.12)). The interaction between MTNR1A gene rs2119882 locus and CLOCK gene rs1801260 locus was associated with the risk of type 2 diabetes (RERI = 1.07, (95% CI, 0.23–1.91); AP = 0.77, (95% CI, 0.36–1.17)). The complex interaction of the MTNR1A-MTNR1B-CLOCK-rotating night shift work model based on the GMDR methods may increase the risk of type 2 diabetes (*P* = 0.011).

**Conclusions:**

Rotating night shift work and rs1387153 variants in MTNR1B were associated with an increased risk of type 2 diabetes among steelworkers. The complex interaction of MTNR1A-MTNR1B-CLOCK-rotating night shift work may increase the risk of type 2 diabetes.

**Supplementary Information:**

The online version contains supplementary material available at 10.1186/s12864-023-09328-y.

## Introduction

The development of type 2 diabetes is a challenging public health problem in many countries, and the incidence of diabetes worldwide is increasing rapidly [1]. It is estimated that by 2025, the number of diabetic patients will increase by 65% to 380 million, which has brought a great burden of disease to the world [2].

Shift work is an irregular or abnormal work system compared with daytime work, such as night work or rotating shifts [3]. This working system generally exists in the service industry, transportation industry, manufacturing industry, and so on, and has many working systems [4]. Disruption of circadian rhythms through shift work directly or indirectly increases the risk of many diseases, such as breast cancer, nonalcoholic fatty liver disease, and type 2 diabetes [5, 6].

Circadian rhythm plays an important role in controlling and maintaining internal balance and adapting to the changes in external environmental conditions [7]. It is reported that many human physiological activities (including the sleep–wake cycle, hormone secretion, periodic changes in body temperature, blood pressure, and blood glucose levels) are related to circadian rhythm [8]. A large number of studies have indicated that the risk of type 2 diabetes would be increased by shift work [9, 10]. Although the specific mechanisms were not yet clear, studies have shown that night shift exposure caused by shift work may disrupt endogenous circadian rhythm, especially the blood glucose levels, inflammatory biomarkers and decrease in insulin sensitivity, which may affect the risk of type 2 diabetes through some direct and indirect pathways [11, 12]. In addition to the external environmental factors such as shift work, CLOCK-controlled (CLOCK) genes, the key gene in the transcriptional translational feedback loops formed by the circadian rhythm system, and melatonin receptor (MTNR) 1A and 1B gene can affect this pathogenic process at the molecular level [13–16]. However, so far, the results of the association between shift work and type 2 diabetes remain inconsistent [17–19], which may be due to different shift evaluation indicators. Therefore, we evaluated rotating night shift work with four indicators, including rotating night shift work, current shift status, duration of night shifts (years) and average frequency of night shifts (night/month). In addition, there is little epidemiological evidence about the interaction between CLOCK, MTNR1A, MTNR1B genes polymorphisms and shift work on type 2 diabetes. Thus, from the perspective of population epidemiology, this study aims to explore the association of rotating night shift work, CLOCK, MTNR1A, MTNR1B genes polymorphisms and their interactions with type 2 diabetes among steelworkers through a case–control study.

## Methods

### Study population

Study participants for this analysis were from the baseline population of a cohort study conducted by the Ministry of science and technology of China. This cohort study was conducted in HBIS Group’s Tangsteel Company in Tangshan, China, and the purpose was to explore the health effects of occupational harmful factors on the human body. The subjects of this study were workers who participated in an occupational health examination in Tangshan Hongci hospital from February to June 2017. Because of the short follow-up period, the number of new cases of type 2 diabetes was far from a nested case–control study. In addition, the funding for our project did not support our testing of the genotypes of all research subjects, so we did not conduct a cross-sectional study. Taking into account human and financial resources as well as available information, we selected a case–control study design based on the baseline data. First, according to the diagnostic criteria of type 2 diabetes, we determined the preliminary case population (961) and control population (6264). The diagnosis of type 2 diabetes was based on the diagnostic principles of the World Health Organization (WHO) in 1999 and the American Diabetes Association’s criteria [20]. The second step was to select cases from the preliminary case population according to the inclusion and exclusion criteria of cases. In the third step, we randomly selected controls from the preliminary control population with SAS 9.4 software according to the inclusion and exclusion criteria of controls. Finally, according to the inclusion and exclusion criteria of cases and controls, 251 cases and 451 controls were selected from 7025 people. In the case group (251), 87 (34.6%) workers who were receiving hypoglycemic therapy or had been diagnosed by the hospital were defined as type 2 diabetes patients, and 164 (65.4%) workers with one or more classic symptoms (excessive thirst, polyuria or frequent urination, weight loss, hunger) plus fasting plasma glucose concentrations of at least 7.0 mmol/L were also defined as type 2 diabetes patients.

#### Inclusion criteria of cases

①Han (The most populous ethnic group in the baseline survey) in-service (At the time of the baseline survey, workers who participated in occupational health examinations included retired, in-service, and those who left for some reasons) workers with type 2 diabetes identified in the baseline data; ②The age is 30 ~ 60 years old; ③The subject must have worked in HBIS Group’s Tangsteel Company in Tangshan for at least three years; ④Those who signed the informed consent form.

#### Exclusion criteria of cases

①The workers with missing blood biochemical data(26), shift information (12), and covariates (18); ②The workers who took some antiretroviral drugs (57), and the workers with cancer (7), excess alcohol intake (63), history of the hepatobiliary disease (59), thyroid disease (76), and renal failure (1) were excluded; ③The workers with other diseases (3) related to circadian clock genes and hormone receptors selected in this study; ④Those who did not sign informed consent were also excluded (9).

#### Inclusion criteria of controls

①Han in-service workers without type 2 diabetes were diagnosed by the same diagnostic criteria in the source population of the cases; ②Matched with the case group according to age (± 5 years); ③The subject must have worked in HBIS Group’s Tangsteel Company in Tangshan for at least three years; ④ From the same workshop as the cases; ⑤ Similar living conditions; ⑥ Those who signed the informed consent form.

#### Exclusion criteria of controls

①The workers with missing blood biochemical data (738), shift information (97), and covariates (137); ②The workers who took some antiretroviral drugs (474), and the workers with cancer (18), excess alcohol intake (122), history of the hepatobiliary disease (452), thyroid disease (409), and renal failure (3) were excluded; ③The workers with other diseases (25) related to circadian clock genes and hormone receptors selected in this study; ④Those who did not sign informed consent were also excluded (56).

$$\begin{array}{c}n=\frac{{\left[{z}_{\propto }\sqrt{2\overline{p }\left(1-\overline{p }\right)}+{z}_{\beta }\sqrt{{p}_{1}\left(1-{p}_{1}\right)+{p}_{0}\left(1-{p}_{0}\right)}\right]}^{2}}{{\left({p}_{1}-{p}_{0}\right)}^{2}}\end{array}$$

$$\begin{array}{c}\overline{p }=\frac{\left({p}_{1}+{p}_{0}\right)}{2}\end{array}$$

$$\begin{array}{c}{p}_{1}=\frac{\left(OR\times {p}_{0}\right)}{\left(1-{p}_{0}+OR\times {p}_{0}\right)}\end{array}$$

*p*_*0:*_ The exposure rate of control group;

*p*_1_: The exposure rate of case group.

In this study, we screened the data according to the National Biotechnology Information Center (NCBI) single nucleotide polymorphism (SNP) database (http://www.ncbi.nlm.nih.gov/SNP). The minimum allele frequency (MAF) of the selected SNP locus was 0.226, significance level α = 0.05, β = 0.10, and expected OR = 2.0. According to the above conditions, the sample size of the case group and the control group was 221. The sample sizes of the case group and control group were 251 and 451, respectively, which met the sample size required for this case–control study. All participants gave informed consent before taking part in this study. This research was approved by the Ethics Committee of the North China University of Science and Technology.

### Assessment of rotating night shift work

In this study, the modern four-crew-three-shift system and the historical three-crew-two-shift system were mainly two kinds of rotating night shift work schedules. Rotating night shift work refers to the work schedule that continuously alternates day shift and night shift for more than 1 year. Workers who have continuously alternated day shift and night shift for more than one year and were still rotating night shift work as of the date of the survey were defined as “current” night shift workers. After more than one year of rotating night shift work, workers who were no longer on rotating night shift work as of the date of the survey were defined as ever-rotating night shift workers. Shift workers whose shift duration didn’t exceed one year and the workers who worked regular working hours at all times were defined as never rotating night shift workers. In the modern four-crew-three-shift system, each group had two-morning shifts (08:00–16:00), two-afternoon shifts (16:00–00:00), two-night shifts (00:00–08:00) and then had 2 days off. In the historical three-crew-two-shift system, each group had a morning shift (08:00–20:00), a night shift (20:00–08:00) and then took 1 day off. The data were collected from face-to-face interviews and then checked with the records of the Tangsteel company. Participants were asked whether they had worked on a shift schedule of alternating day and night shifts (3 or more hours of working time between 00:00 and 05:00) [21] during their employment. If yes, they were asked about their current shift status (ever, current), rotating night shift work schedules, schedule arrangement, start date and end date of each schedule. In this study, different exposure indicators were used to evaluate rotating night shift work, such as rotating night shift work, current shift status, duration of night shifts (years), and average frequency of night shifts (nights/month). The specific evaluation and calculation of shift indicators were shown in the supplementary file.

#### Blood tests and assessment of covariates

Subjects were asked to fast for 8 h before veinous blood from the anterior elbow can be drawn. Blood samples were collected and centrifuged immediately at room temperature (3000 r/min, 15 min). All blood samples were tested in the central laboratory of Tangshan Hongci Hospital using automatic biochemical analyzers (mindray, BS-800, China) within four hours.

After several pre-surveys, the questionnaire of the study has been revised repeatedly, and the survey was conducted face to face with participants by uniformly trained personnel. In the questionnaire, the data on sex, age, family disease history, smoking, drinking, physical exercise, diet, sleep and occupational exposure factors (including high temperature, noise, dust, and carbon monoxide (CO)) were also collected. In addition, we measured the height and weight of the participants. The specific assessment of these covariates was shown in the supplementary file.

#### Detection of CLOCK, MTNR1A and MTNR1B genes

DNA in blood was extracted by a blood genomic DNA extraction kit (Beijing Tiangen Biochemical Technology Co., Ltd). After genomic DNA extraction, its purity and concentration were measured by spectrophotometer. According to the gene information of the Han population in Ensembl, 1000 Genomes Project database and NCBI database, Tag SNPs were screened by the Tagger algorithm of haplotype analysis software Haploview 4.2. The conditions were that the MAF of tag SNPs in the Chinese population was ≥ 8% and the linkage disequilibrium coefficient (pairwise correlation coefficient, r^2^) with other SNPs in the region was greater than 0.8. When multiple SNPs met the above two points at the same time, the SNPs related to type 2 diabetes that have been identified by the genome-wide association study (GWAS) were preferred; Functional regions, such as promoter region, 3’ untranslated region, URT, 5’ UTR and exon region, were considered next; When multiple loci were in the functional region at the same time or all loci were in the nonfunctional region, the loci with the strongest linkage degree were preferred. After a comprehensive analysis, 3 SNPs were finally selected.

The primers in this study were designed by Shanghai Biotechnology Co., Ltd, and the appropriate primers were selected according to the Rolling circle replication (PCR) and enzyme digestion conditions. Thus, the primer information used in this study was shown in table S1. (Table S1, Supplementary file). Before PCR amplification, the synthesized target SNPs primers were fully dissolved in pure water and stored in a refrigerator at—20℃. The PCR reaction system used in the experiment and the PCR reaction conditions of the target SNPs were shown in Table S2 and Table S3, respectively. (Tables S2, and S3, Supplementary file).

**Fig. 1:**
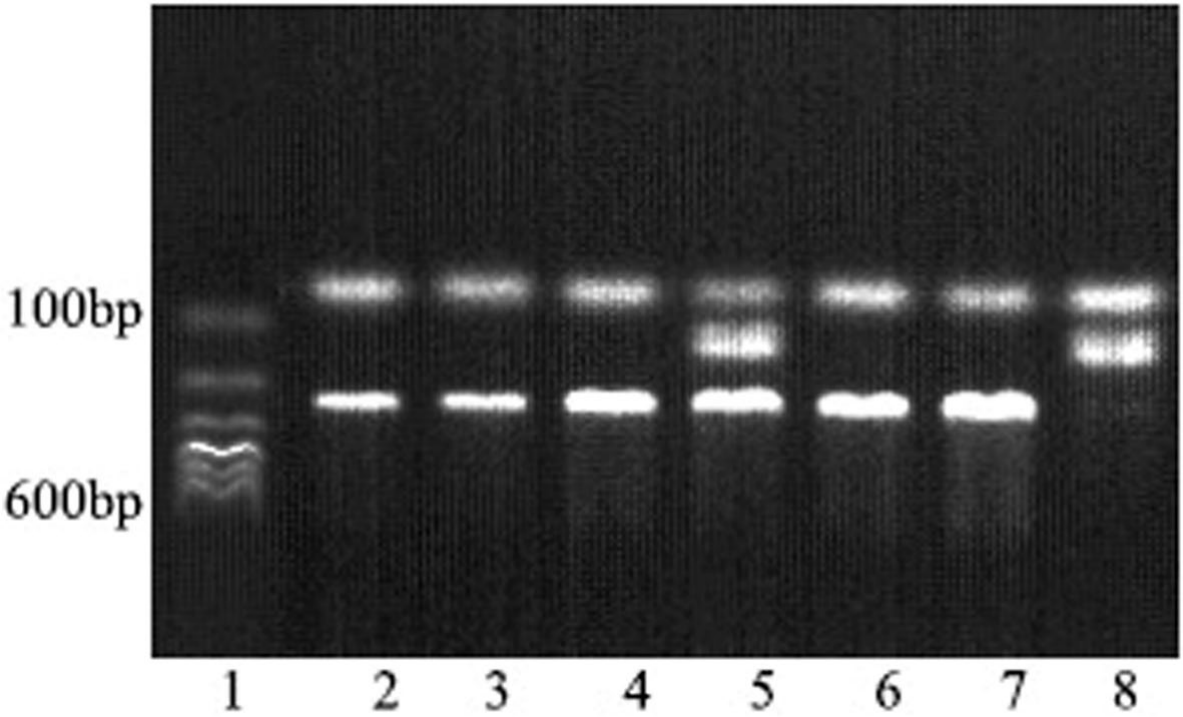
The electrophoretic gel results of rs1801260 locus for CLOCK gene

**Fig. 2:**
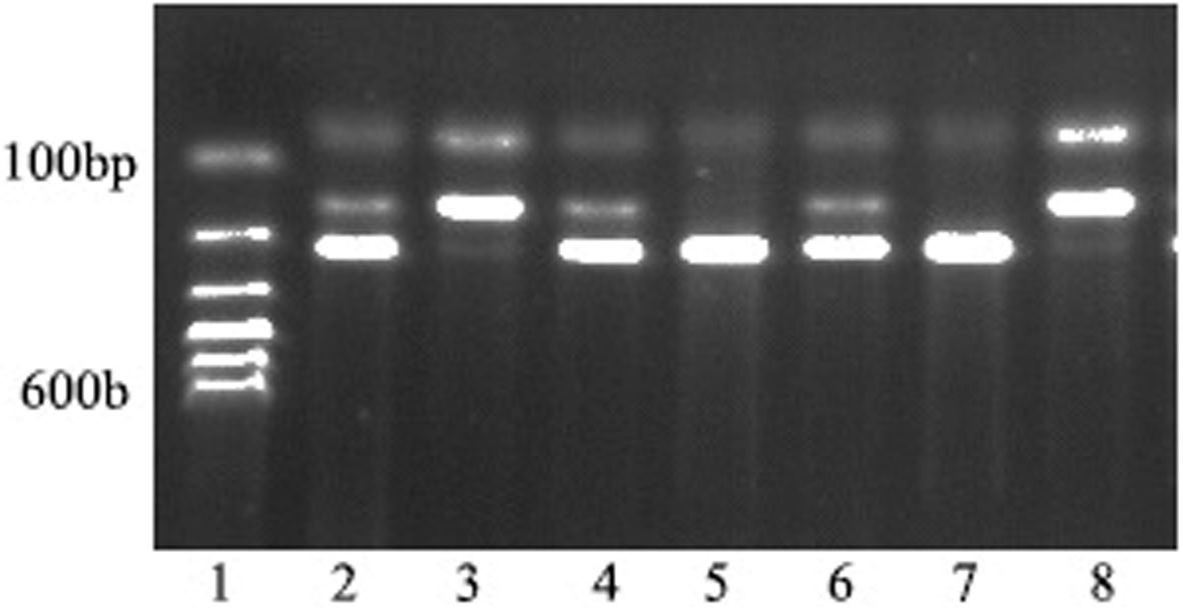
The electrophoretic gel results of rs2119882 locus for MTNR1A gene

**Fig. 3:**
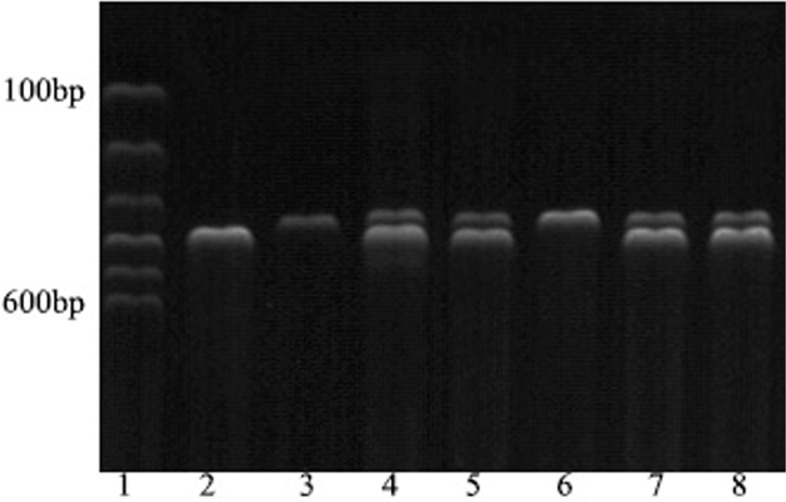
The electrophoretic gel results of rs1387153 locus for MTNR1B gene

According to the PCR amplification of target SNPs and the size of the restriction fragment, the corresponding agarose gel was prepared, and rolling circle replication-restriction fragment length polymorphism (PCR–RFLP) was used for restriction enzyme digestion. (Enzyme digestion reaction system and conditions see Table S4, Supplementary file). After electrophoresis, the fragment size of enzyme digestion products in the electrophoresis map was determined by a UV analyzer. Finally, the gel image analysis system was used to identify and photograph the genotypes. (The results of enzyme digestion were shown in Figs. 1, 2, and 3 and the original electrophoretic gel results were shown in Figs. S1, S2, and S3, Supplementary file).

#### Statistical analyses

Continuous variables were represented by means ± standard deviation. If the continuous variables met the normality and homogeneity of variance, the differences between the case group and the control group were compared by the independent-sample T test. If not, the differences between groups were compared by Wilcoxon’s rank-sum test. Categorical variables were represented by the number of individuals (%), and the differences between groups were compared by chi-square tests. The Chi-square test was used to determine whether the survey object was a random sample in the target population, according to Hardy–Weinberg equilibrium (HWE). The logistic regression was used to explore the effect of CLOCK, MTNR1A, MTNR1B genes polymorphisms and rotating night shift work on the risk of type 2 diabetes. Previous studies [19, 22, 23] have shown that sleep and BMI were mediating roles between shift and diabetes, so these two factors were excluded when adjusting for confounding factors. The effect of gene–gene interactions and genes-rotating night shift work interactions based on the multiplicative and additive model on type 2 diabetes were explored by logistic regression, generalized multifactor dimensionality (GMDR), and additive interaction model improved by Andersson et al. [24]. Analyses were conducted with Statistical Package for the Social Sciences (SPSS for Windows, version 19.0, SPSS Inc, Chicago, IL, USA) software and Statistical Analysis System Version 9.4 (SAS, Institute, Raleigh, NC) with a two-sided significance threshold of *P* < 0.05. Bonferroni correction was used for the correction of multiple comparisons.

## Results

### Characterization of study population

The basic characteristics of the case group and control group were shown in Table 1. There was no difference in sex among different groups. However, the age of the case group (47.6 ± 7.6 years old) was higher than that of the control group (45.1 ± 8.2 years old). In terms of lifestyle, smoking status and average sleep duration differed by group, and the case group seemed to have less average sleep duration. In terms of occupational hazards, the compositions of people exposed to high temperature, noise and rotating night shift work were different by type 2 diabetes status, and the composition ratio of the number of rotating night shift workers in the case group was higher. It was worth noting that there was no difference in the family history of type 2 diabetes between the case group and the control group.

**Table 1:** Basic characteristics of case group and control group

Characteristics	Control group	Case group	χ^2^ /*Z*	*P* value
	*n* = 451	*n* = 251		
Sex, n (%)			1.276	0.259
Male	440 (97.6)	248 (98.8)		
Female	11 (2.4)	3 (1.2)		
Smoking status, n (%)			6.04	0.049
Never	175 (38.8)	76 (30.2)		
Ever	24 (5.3)	20 (8.0)		
Current	252 (55.9)	155 (61.8)		
Drinking status, n (%)			3.145	0.207
Never	257 (57.0)	130 (51.8)		
Ever	10 (2.2)	3 (1.2)		
Current	184 (40.8)	118 (47.0)		
Physical activity (MET- hours/week), n (%)			1.205	0.547
Low	11 (2.4)	4 (1.6)		
Middle	30 (6.7)	21 (8.4)		
High	410 (90.9)	226 (90.0)		
Hypertension, n (%)			2.294	0.130
No	323 (71.6)	166 (66.1)		
Yes	128 (28.4)	85 (33.9)		
Dyslipidaemia, n (%)			0.409	0.523
No	261 (57.9)	139 (55.4)		
Yes	190 (42.1)	112 (44.6)		
Liver dysfunction, n(%)			3.429	0.064
No	376 (83.4)	195 (77.7)		
Yes	75 (16.6)	56 (22.3)		
Family history of diabetes			1.788	0.181
No	405 (89.8)	217 (86.5)		
Yes	46 (10.2)	34 (13.5)		
Renal dysfunction, n (%)			0.396	0.350
No	382 (84.7)	217 (86.5)		
Yes	69 (15.3)	34 (13.5)		
BMI (kg/m^2^), n (%)			0.103	0.950
< 24	172 (38.1)	98 (39.0)		
24–27	220 (48.8)	122 (48.6)		
≥ 28	59 (13.1)	31 (12.4)		
High temperature, n (%)			2.075	0.013
No	225 (49.9)	111 (44.2)		
Yes	226 (50.1)	140 (55.8)		
Noise, n (%)			3.917	0.048
No	2 (0.4)	5 (2.0)		
Yes	449 (99.6)	246 (98.0)		
Dust, n (%)			2.517	0.113
No	80 (17.7)	33 (13.1)		
Yes	371 (82.3)	218 (86.9)		
Rotating night shift work			11.389	0.001
Never	105 (23.3)	32 (12.7)		
Yes	346 (76.7)	219 (87.3)		
CO, n (%)			1.546	0.214
No	207 (45.9)	103 (41.0)		
Yes	244 (54.1)	148 (59.0)		
Age (years)	45.1 ± 8.2	47.6 ± 7.6	3.831	< 0.001
Average sleep duration (h)	6.8 ± 1.2	6.5 ± 1.4	2.582	0.010
DASH Score	21.4 ± 2.2	21.7 ± 2.3	1.727	0.084

Values were presented as mean ± standard deviation or as number of individuals (%). *P*-values were from chi-square tests for categorical variables and Wilcoxon testing or one-way ANOVA for continuous variables

*CO* carbon monoxide, *DASH* dietary approaches to stop hypertension, *MET* metabolic equivalent of task, *BMI* body mass index

### Main effect of rotating night shift work

The *P* values for trend and *OR* between different exposure metrics of night shift work and type 2 diabetes had been given in Table 2. The association between rotating night shift work, current shift status, duration of night shifts, and average frequency of night shifts and increased risk of type 2 diabetes were statistically significant before and after adjustment for confounders. Compared with those who never night shift work, the groups with the rotating night shift work, ever night shift work and current night shift work, had an *OR* of type 2 diabetes of 2.00, (95% CI, 1.28–3.11), 1.86, (95% CI, 1.12–3.08) and 2.08, (95% CI, 1.31–3.30) after adjusting for age, sex, smoking status, drinking status, physical activity, dietary approaches to stop hypertension (DASH) score, dyslipidemia, hypertension, liver dysfunction, renal dysfunction and exposure to occupational hazards (high temperature, noise, dust and CO), respectively. Compared with never night shift, the duration of night shifts of 1–10 years, 10–20 years, 20–30 years and more than 30 years were associated with a higher risk of type 2 diabetes after adjusting for potential confounding factors. Then, compared with never night shift, the average frequency of night shifts of 3–8 nights/month and more than 8 nights/month were associated with a higher risk of type 2 diabetes after adjusting for potential confounding factors. Furthermore, the risk of type 2 diabetes was increasing with the increasing years and frequency of night shifts after adjusting for potential confounding factors, and the *ORs* (95% CIs) were 1.15 (95% CIs, 1.01–1.30) and 1.39 (95% CIs, 1.15–1.68), respectively.

**Table 2:** Odds ratios for type 2 diabetes according to different exposure metrics of night shift work

Exposure metrics	Control group,n(%)	Case group, n(%)	Model 1	Model 2
			*OR* (95% CI)	*OR* (95% CI)
Rotating night shift work				
Never	105 (23.3)	32 (12.7)	1.00 (ref)	1.00 (ref)
Yes	346 (76.7)	219 (87.3)	**2.08 (1.35–3.19)**	**2.00 (1.28–3.11)**
Current shift status				
Never	105 (23.2)	32 (12.7)	1.00 (ref)	1.00 (ref)
Ever	124 (27.5)	69 (27.5)	**1.83 (1.12–2.99)**	**1.86 (1.12–3.08)**
Current	222 (49.3)	150 (59.8)	**2.22 (1.42–3.47)**	**2.08 (1.31–3.30)**
Duration of night shifts (years)				
Never	105 (23.2)	32 (12.7)	1.00 (ref)	1.00 (ref)
1–10	99 (22.0)	49 (19.5)	1.62 (0.96–2.74)	**1.80 (1.04–3.12)**
10–20	77 (17.1)	47 (18.7)	**2.00 (1.17–3.43)**	**2.30 (1.31–4.04)**
20–30	118 (26.2)	87 (34.8)	**2.42 (1.49–3.92)**	**2.23 (1.35–3.67)**
> 30	52 (11.5)	36 (14.3)	**2.27 (1.27–4.06)**	**1.51(0.82–2.80)**
*OR* (95% CI) for trend			**1.24 (1.10–1.39)**	**1.15 (1.01–1.30)**
Average frequency of night shifts (nights/month)				
Never	105 (23.2)	32 (12.7)	1.00 (ref)	1.00 (ref)
< 3	115 (25.5)	62 (24.7)	**1.77(1.07–2.92)**	1.64 (0.97–2.76)
3–8	206 (45.8)	134 (53.4)	**2.13 (1.36–3.35)**	**2.10 (1.32–3.34)**
> 8	25 (5.5)	23 (9.2)	**3.02 (1.51–6.02)**	**2.72 (1.33–5.57)**
*OR* (95% CI) for trend			**1.41 (1.17–1.69)**	**1.39 (1.15–1.68)**

Model 1: univariate analysis. Model 2: adjusted for age, sex, smoking status, drinking status, physical activity, DASH score, dyslipidaemia, hypertension, liver dysfunction, renal dysfunction and exposure to occupational hazards (high temperature, noise, dust and carbon monoxide (CO)) in each exposure metric. DASH, dietary approaches to stop hypertension. Value in bold: it indicates that it is statistically significant

### Main effect of CLOCK, MTNR1A, MTNR1B genes polymorphisms

Without considering shift work, the main effects of gene polymorphism have been explored in univariate and multivariate models. The results of association analysis between MTNR1B gene rs1387153 locus and type 2 diabetes among steelworkers were shown in Table 3. The univariate and multivariate results suggested that under the codominant model, workers with the TT gene had an increased risk of type 2 diabetes compared with those with CC genotype, and ORs, (95% CIs) were 2.08, (95% CI, 1.33–3.25) and 1.96, (95% CI, 1.22–3.14), respectively. Under the recessive model, compared with CC or CT genotype workers, TT genotype workers had a higher risk of type 2 diabetes and ORs, (95% CIs) were 1.94, (95% CI, 1.31–2.87) and 1.78, (95% CI, 1.18–2.68) before and after adjusting for confounders. Then, under the Log-additive model, univariate and multivariate analysis showed that with the increase in the number of T alleles, the risk of type 2 diabetes increased by 1.40, (95% CI, 1.12–1.75) and 1.37, (95% CI, 1.09–1.73), respectively. (Table 3).

**Table 3:** The association between MTNR1B gene rs1387153 locus and type 2 diabetes among steelworkers

Gene	Gene Model	Genotype	Control group, n (%)	Case group, n(%)	Model 1	Model 2
					***OR*** ** (95% CI)**	***OR*** ** (95% CI)**
MTNR1B gene rs1387153 locus	Codominant	C/C	157 (34.8)	72 (28.7)	1.00 (ref)	1.00 (ref)
		C/T	230 (51.0)	118 (47.0)	1.12 (0.78–1.60)	1.18 (0.81–1.71)
		T/T	64 (14.2)	61 (24.3)	**2.08 (1.33–3.25)**	**1.96 (1.22–3.14)**
	Dominant	C/C	157 (34.8)	72 (28.7)	1.00 (ref)	1.00 (ref)
		C/T-T/T	294 (65.2)	179 (71.3)	1.33 (0.95–1.86)	1.36 (0.96–1.93)
	Recessive	C/C–C/T	387 (85.8)	190 (75.7)	1.00 (ref)	1.00 (ref)
		T/T	64 (14.2)	61 (24.3)	**1.94 (1.31–2.87)**	**1.78 (1.18–2.68)**
	Overdominant	C/C-T/T	221 (49.0)	133 (53.0)	1.00 (ref)	1.00 (ref)
		C/T	230 (51.0)	118 (47.0)	0.85 (0.63–1.16)	0.92 (0.66–1.27)
	Log-additive	–-	–-	–-	**1.40 (1.12–1.75)**	**1.37 (1.09–1.73)**

Model 1: univariate analysis. Model 2: adjusted for age, sex, smoking status, drinking status, physical activity, DASH score, dyslipidaemia, hypertension, liver dysfunction, renal dysfunction and exposure to occupational hazards (high temperature, noise, dust and carbon monoxide (CO)) in each exposure metric. DASH, dietary approaches to stop hypertension. Value in bold: it indicates that it is statistically significant

In addition, the results of association analysis between MTNR1A gene rs2119882 locus, CLOCK gene rs1801260 locus and type 2 diabetes among steelworkers were shown in table S5. Under all models, no statistically significant association was found between MTNR1A gene rs2119882 locus, CLOCK gene rs1801260 locus and type 2 diabetes. (Table S5, Supplementary file).

### Gene–gene interactions and gene-environment interactions

The multiplicative and additive interaction between rotating night shift work and genes on the risk of type 2 diabetes were shown in Table 4. Compared with workers who never night shift work and carry rs2119882 locus TT genotype, workers who rotate night shift work and carry rs2119882 locus TT genotype had an increased risk of type 2 diabetes, and multivariable-adjusted OR, (95% CI) was 2.36, (95% CI, 1.14–4.87). The multiplicative and additive interaction between rotating night shift work and rs2119882 locus on the risk of type 2 diabetes was not statistically significant. However, we documented a significant additive interaction between rotating night shift work and rs1387153 on type 2 diabetes, with the relative excess risk due to interaction (RERI) and attributable proportions (AP) of 0.98, (95% CI, 0.40–1.55) and 0.60, (95% CI, 0.07–1.12), respectively. Then, the multivariable-adjusted OR for type 2 diabetes among steelworkers with rotating night shift work and with rs1801260 locus TT genotype was 2.08, (95% CI, 1.27–3.38). The joint effect of rotating night shift work and rs1801260 locus CT or CC genotype were statistically significant, and ORs, (95% CIs) were 2.85, (95% CI, 1.56–5.18) and 2.60, (95% CI, 1.40–4.84) before and after adjusting for confounders. However, the multiplicative and additive interactions between them were not significant. (Table 4) In addition, the impact of the multiplicative interactions between other shift indicators and genes on the risk of type 2 diabetes has also been explored in the Supplementary file Tables S6, S7, and S8. The significant multiplicative interactions between current shift work (Table S6, Supplementary file), the duration of night shifts (Table S7, Supplementary file), and rs1387153 on type 2 diabetes were documented. The interactions between the average frequency of night shifts and the three loci were not statistically significant. (Table S8, Supplementary file).

**Table 4:** Multiplicative and additive interaction between rotating night shift work and gene on risk of type 2 diabetes

Genotype	Factors	Control group, n	Case group, n	*OR* (95% CI)		*P* for multiplicative interaction	
				Model 1	Model 2	Model 1	Model 2
rs2119882	Rotating night shift work					0.652	0.562
T/T	No	38	12	1.00 (ref)	1.00 (ref)		
C/T-C/C	No	67	20	0.95 (0.42–2.14)	1.03 (0.44–2.39)		
T/T	Yes	115	86	**2.37 (1.17–4.80)**	**2.36 (1.14–4.87)**		
C/T-C/C	Yes	231	133	1.82 (0.92–3.61)	1.85 (0.92–3.75)		
	RERI			-0.49 (-1.81–0.83)	-0.54 (-1.92–0.85)		
	AP			-0.27 (-0.89–0.36)	-0.29 (-0.94–0.36)		
rs1387153	Rotating night shift work					0.069	0.054
C/C	No	27	11	1.00 (ref)	1.00 (ref)		
C/T-T/T	No	78	21	0.66 (0.22–1.55)	0.62 (0.26–1.47)		
C/C	Yes	130	61	1.15 (0.54–2.47)	1.04 (0.48–2.29)		
C/T-T/T	Yes	216	158	1.80 (0.87–3.73)	1.64 (0.77–3.45)		
	RERI			**1.15 (0.54–2.47)**	**0.98 (0.40–1.55)**		
	AP			**0.66 (0.28–1.55)**	**0.60 (0.07–1.12)**		
rs1801260	Rotating night shift work					0.729	0.639
T/T	No	92	26	1.00 (ref)	1.00 (ref)		
C/T-C/C	No	13	6	1.63 (0.57–4.72)	1.66 (0.56–4.93)		
T/T	Yes	295	178	**2.14 (1.33–3.43)**	**2.08 (1.27–3.38)**		
C/T-C/C	Yes	51	41	**2.85 (1.56–5.18)**	**2.60 (1.40–4.84)**		
	RERI			0.07 (-1.97–2.12)	-0.14 (-2.23–1.95)		
	AP			0.03 (-0.69–0.74)	-0.05 (-0.87–0.76)		

Model 1: univariate analysis. Model 2: adjusted for age, sex, smoking status, drinking status, physical activity, DASH score, dyslipidaemia, hypertension, liver dysfunction, renal dysfunction and exposure to occupational hazards (high temperature, noise, dust and carbon monoxide (CO)). *DASH* dietary approaches to stop hypertension, *RERI* Relative excess risk due to interaction, *AP* Attributable proportion. Value in bold: it indicates that it is statistically significant

Equally important, the gene–gene interactions were also shown in Table S9. The univariate and multivariate results suggested that the additives and multiplicative interactions between rs1801260 locus and rs2119882 locus on type 2 diabetes were statistically significant. However, the results of cross-classification analysis showed that their joint effects were not statistically significant. (Table S9, Supplementary file) So, we further explored the association between their joint effect and type 2 diabetes among steelworkers stratified by rs2119882 and rs1801260. Compared with the population carrying the rs2119882 CT or CC genotype and the rs1801260 TT genotype, the population carrying the rs2119882 CT or CC genotype and the rs1801260 CT or CC genotype had an increased risk of type 2 diabetes, with a multivariable-adjusted OR, (95% CI) was 1.87, (95% CI, 1.07–3.28). Compared with the population carrying the rs2119882 TT genotype and the rs1801260 CT or CC genotype, the population carrying the rs2119882 CT or CC genotype and the rs1801260 CT or CC genotype had an increased risk of type 2 diabetes, with a multivariable-adjusted OR, (95% CI) was 3.89, (95% CI, 1.22–12.42). (Table S10, Supplementary file) Additionally, other additive and multiplicative gene–gene interactions have not been observed. (Table S9, Supplementary file).

Furthermore, Genetic and environmental factors with additive or multiplicative interactions between them were analyzed by stratification. Only in the population carrying the rs1387153 locus CT or TT genotype, the association between rotating night shift work and increased risk of type 2 diabetes was statistically significant. Similarly, we found that compared with workers carrying rs1801260 locus TT genotype, the risk of type 2 diabetes with rs1801260 locus CT or CC genotype workers increased among workers carrying rs2119882 locus CT or CC genotype. (Table S11, Supplementary file).

Finally, we conducted the gene-environment interactions through GMDR in Table 5. After adjusting for confounding factors, the cross-validation consistency of the best four-factor model reached 100% (10/10), and this four-factor model was statistically significant. Furthermore, the training and test balanced accuracy of this four-factor model reached 0.6137 and 0.5574, respectively. And other models had no statistical significance, so the MTNR1A-MTNR1B-CLOCK-rotating night shift work model was selected. (Fig. S4, Supplementary file).

**Table 5:** The interaction models based on the GMDR

Model	Cross-validation consistency	Training balanced accuracy	Test balanced accuracy	*P*
Shift	7/10	0.5543	0.5319	0.172
MTNR1B-Shift	8/10	0.5777	0.5457	0.377
MTNR1B-CLOCK-Shift	5/10	0.5924	0.5171	0.172
MTNR1A-MTNR1B-CLOCK-Shift	10/10	0.6137	0.5574	0.011

Adjusted for age, sex, smoking status, drinking status, physical activity, DASH score, dyslipidaemia, hypertension, liver dysfunction, renal dysfunction and exposure to occupational hazards (high temperature, noise, dust and carbon monoxide (CO))

*DASH* dietary approaches to stop hypertension, *BMI* body mass index, *RERI* Relative excess risk due to interaction, *AP* Attributable proportion; shift: rotating night shift work

In addition, although no statistical significance was found in the validation set, in the best interaction model, workers with homozygous mutations had a significantly higher risk of type 2 diabetes than those with homozygous wild-type in the total dataset. (Table S12, Supplementary file).

### Sensitivity analyses

The purpose of the stratified analysis was to explore whether the association between rotating night shift work and type 2 diabetes was still stable among workers who ever or currently rotated night shift work. Univariate and multivariate results showed that the association between duration of night shifts, the average frequency of night shifts and type 2 diabetes were both significant, no matter in which stratification. Compared with those who never night shift work, the risk of type 2 diabetes was higher with the longer duration of night shifts among ever night shift workers (*P* = 0.009). And compared with those who never night shift work, the risk of type 2 diabetes was higher with the higher average frequency of night shifts among current night shift workers (*P* = 0.001). (Table S13, Supplementary file).

In conclusion, compared with Table 2, the results of sensitivity analysis were still robust. Even after stratification, the relationship between the duration and average frequency of night shifts and type 2 diabetes were still statistically significant, which was consistent with the findings in Table 2 to a large extent.

## Discussion

The present analysis showed that rotating night shift work and MTNR1B gene rs1387153 locus were associated with increased risk of type 2 diabetes, and the association between rotating night shift work and risk of type 2 diabetes appeared to be modified by MTNR1B gene rs1387153 locus. The results showed that there were significant gene-environment interactions among rotating night shift work, current shift status, the duration of night shifts and MTNR1B gene rs1387153 locus on type 2 diabetes. Moreover, there was a positive gene–gene interaction between CLOCK gene rs1801260 locus and MTNR1A gene rs2119882 locus on type 2 diabetes. At present, this study found that people who carry both the rs1801260 locus CT or CC genotype and the rs2119882 locus CT or CC genotype had a higher risk of type 2 diabetes. The complex interaction of the MTNR1A-MTNR1B-CLOCK-rotating night shift work model could significantly increase the risk of type 2 diabetes.

Although the results of the association between shift work and the risk of type 2 diabetes were inconsistent, the results generally agreed with existing evidence from some cohort studies [17, 25, 26]. There were several reasons for the inconsistent results of previous studies, such as different assessments of shift work and different study populations. In this study, we evaluated shift work through four indicators, such as rotating night shift work (yes or never), current shift status (never ever and current), duration of night shifts (years) and average frequency of night shifts (nights/month). Although the grouping of the duration of night shifts was different, this research was consistent with the findings of Pan A [27] and Vimalananda VG [28], which showed that the increase in the duration of night shifts may increase the risk of type 2 diabetes. An early paper found that the positive association between shift working years and type 2 diabetes was entirely mediated by weight [19]. In contrast, a multivariate study also showed that even after adjusting for BMI, the association between years of night-shift work and type 2 diabetes was still significant [29]. The results of this multivariate study showed that the association was significant even after adjusting for occupational hazards. A study based on the UK Biobank showed that working more night shifts per month was associated with a higher incidence rate of type 2 diabetes than never shift workers [30]. The results of this study suggested that working more night shifts per month increased the risk of type 2 diabetes, which was consistent with this study to some extent. This indicated that the circadian rhythm disorder caused by long duration and high-frequency night shift work should be taken into consideration in exploring the relationship between type 2 diabetes and shift work.

The CLOCK, MTNR1A and MTNR1B genotype frequencies of the subjects in this study were in accordance with the Hardy–Weinberg equilibrium. The findings of the study provide evidence that encoding the MTNR1B gene may play a role in type 2 diabetes etiology, as the MTNR1B gene was associated with type 2 diabetes risk in this data. Previous studies tend to focus more on the rs10830963 locus in the MTNR1B gene [31–33]. In contrast, this study suggested that the MTNR1B gene rs1387153 locus was associated with the increased risk of type 2 diabetes, which was supported by some studies [34, 35]. In addition, this study found a significant interaction between the MTNR1B gene rs1387153 locus and rotating night shift work on type 2 diabetes, which was basically consistent with the results of a laboratory study [11]. We also found a significant SNP-SNP interaction between rs2119882 and rs1801260. Furthermore, we found the association between high-dimensional interaction in the MTNR1A-MTNR1B-CLOCK-rotating night shift work model on type 2 diabetes through GMDR. To our knowledge, there are few studies on the high-dimensional interaction between circadian clock genes and melatonin receptor genes and rotating night shift work on type 2 diabetes based on GMDR. The results suggested that these interactions affect may contribute to the risk of type 2 diabetes in some complex biological mechanisms.

The potential complex biological mechanisms under the interaction model need to be further studied in the future, but some correlations seem to be explained. At the molecular level, a series of interacting clock proteins maintain the circadian rhythm of the body through transcriptional translational feedback loops [36, 37]. As a core clock gene, the CLOCK gene plays an important role in the initiation and maintenance of the circadian rhythm system [38, 39]. In fact, the existence of circadian clock genes in pancreatic β-cells has been proved by many studies [40, 41]. Melatonin inhibits insulin secretion by β-cells [14], which suggested a link between abnormal melatonin signaling and the risk of type 2 diabetes. MTNR1A and MTNR1B are the main melatonin receptors in humans [42], and abnormal variants of them would trigger aberrant changes in melatonin that may contribute to the pathogenesis of type 2 diabetes [43]. In addition to the biological mechanism at the molecular level, the balance of circadian rhythm can be broken through the external environment. For example, shift workers were exposed to artificial lights at night, which can also trigger aberrant changes in melatonin [44]. Thus, the risk of type 2 diabetes may be increased by their interaction.

## Strengths and limitations of study

In this study, we have considered many variables, and we have checked some variables in Tangsteel company to ensure their accuracies, such as shift schedule (including current shift status, duration of night shifts and average frequency of night shifts) and occupational hazards (including high temperature, noise, dust and CO). To our knowledge, this study is the first study to investigate the interaction between circadian rhythm-related genes and shift work on type 2 diabetes among steelworkers using the log-linear model and GMDR method. The study also has certain limitations. First, this study was a case–control study, unable to determine the causal relationship between shift and type 2 diabetes. Secondly, part of the cases was defined according to the fasting blood glucose value only once, which may bring some bias to the results to a certain extent. Thirdly, some of the information obtained through face-to-face interviews may be inaccurate, such as family history of type 2 diabetes, smoking and drinking. Fourth, there were some selection biases in our research. We excluded the subjects with missing covariates and shift information when selecting cases and controls, which may cause some bias in the results; the occurrence of type 2 diabetes is a long process, and those who were about to have type 2 diabetes may be wrongly considered as non-cases; the participants selected for the study may differ from those not selected in some characteristics, such as those who were older and had a longer duration of night shifts were not selected for the study, while those who were relatively younger were selected for the study, which would cover the relationship between shift work and type 2 diabetes to some extent. Fifth, the information about the circulation level of other markers was missing in this study, which limited the interpretation of the results to a certain extent and it should be fully considered in future research. Finally, the subjects were from steel company, and there were some limitations in the extrapolation of the results.

## Conclusions

Rotating night shift work and rs1387153 variants in MTNR1B were associated with an increased risk of type 2 diabetes even after multivariate adjustment. These findings were consistent with some previous studies, and provide support for these associations. The complex interaction of the MTNR1A-MTNR1B-CLOCK-rotating night shift work model could significantly increase the risk of type 2 diabetes. The findings of the study of the complex gene-environment interactions may be helpful in developing effective prevention strategies to prevent type 2 diabetes. Further research is needed to confirm our findings and clarify the specific biological mechanism.

## Supplementary Information

**Additional file 1.**

## Data Availability

All of the datasets referenced in this study can be obtained upon reasonable request to the corresponding authors.

## Abbreviations

OR: Odds ratios
CI: Confidence intervals
CLOCK: CLOCK-controlled
MTNR: Melatonin receptor
GMDR: Generalized multifactor dimensionality
CO: Carbon monoxide
IPAQ: International Physical Activity Questionnaire
DASH: Dietary approaches to stop hypertension
MET: Metabolic equivalent of task
BMI: Body mass index
eGFR: Estimated glomerular filtration rate
SPSS: Statistical Package for the Social Sciences
SAS: Statistical Analysis System
RERI: Relative excess risk due to interaction
AP: Attributable proportions
WHO: World Health Organization
NCBI: National Biotechnology Information Center
SNP: Single nucleotide polymorphism
GWAS: Genome wide association study
RCR: Rolling circle replication
MAF: Minimum allele frequency
PCR–RFLP: Rolling circle replication-restriction fragment length polymorphism
HWE: Hardy–Weinberg equilibrium

## References

[1] Zimmet P, Alberti KG, Shaw J (2001). Global and societal implications of the diabetes epidemic. Nature.

[2] Van Dieren S, Beulens JW, Van Der Schouw YT (2010). The global burden of diabetes and its complications: an emerging pandemic. Eur J Cardiovasc Prev Rehabil.

[3] Vyas MV, Garg AX, Iansavichus AV (2012). Shift work and vascular events: systematic review and meta-analysis. BMJ.

[4] Buchvold HV, Pallesen S, Waage S (2018). Shift work schedule and night work load: effects on body mass index-a four-year longitudinal study. Scand J Work Environ Health.

[5] Wang XS, Armstrong ME (2011). Shift work and chronic disease: the epidemiological evidence. Occup Med (Lond).

[6] Zhang S, Wang Y, Wang Z (2020). Rotating night shift work and non-alcoholic fatty liver disease among steelworkers in China: a cross-sectional survey. Occup Environ Med.

[7] Loudon AS (2012). Circadian biology: a 2.5 billion year old clock. Curr Biol.

[8] Bechtold DA, Gibbs JE, Loudon ASI (2010). Circadian dysfunction in disease[J]. Trends Pharmacol Sci.

[9] Morikawa Y, Nakagawa H, Miura K (2005). Shift work and the risk of type 2 diabetes among Japanese male factory workers. Scand J Work Environ Health.

[10] Gan Y, Yang C, Tong X (2015). Shift work and type 2 diabetes: a meta-analysis of observational studies. Occup Environ Med.

[11] Scheer FA, Hilton MF, Mantzoros CS (2009). Adverse metabolic and cardiovascular consequences of circadian misalignment. Proc Natl Acad Sci U S A.

[12] Cappuccio FP, D'elia L, Strazzullo P (2010). Quantity and quality of sleep and incidence of type 2 diabetes: a systematic review and meta-analysis. Diabetes Care.

[13] Ramracheya RD, Muller DS, Squires PE (2008). Function and expression of melatonin receptors on human pancreatic islets. J Pineal Res.

[14] Von Gall C, Stehle JH, Weaver DR (2002). Mammalian melatonin receptors: molecular biology and signal transduction. Cell Tissue Res.

[15] Morcuende JA, Minhas R, Dolan L, et al. Allelic variants of human melatonin 1A receptor in patients with familial adolescent idiopathic scoliosis. Spine (Phila Pa 1976). 2003;28(17):2025–8; discussion 2029. 10.1097/01.BRS.0000083235.74593.49. 10.1097/01.BRS.0000083235.74593.49 12973153

[16] Karthikeyan R, Marimuthu G, Spence DW (2014). Should we listen to our clock to prevent type 2 diabetes mellitus?. Diabetes Res Clin Pract.

[17] Eriksson AK, van den Donk M, Hilding A (2013). Work stress, sense of coherence, and risk of type 2 diabetes in a prospective study of middle-aged Swedish men and women. Diabetes Care.

[18] Morikawa Y, Nakagawa H, Miura K (2005). Shift work and the risk of diabetes mellitus among Japanese male factory workers. Scand J Work Environ Health.

[19] Kroenke CH, Spiegelman D, Manson J (2007). Work characteristics and incidence of type 2 diabetes in women. Am J Epidemiol.

[20] Expert Committee on the Diagnosis and Classification of type 2 diabetes. Report of the expert committee on the diagnosis and classification of type 2 diabetes. Diabetes Care. 2003;26 Suppl 1:S5-S20. 10.2337/diacare.26.2007.s5 10.2337/diacare.26.2007.s5 12502614

[21] Stevens RG, Hansen J, Costa G (2011). Considerations of circadian impact for defining 'shift work' in cancer studies: IARC Working Group Report. Occup Environ Med.

[22] Hulsegge G, Proper KI, Loef B (2021). The mediating role of lifestyle in the relationship between shift work, obesity and diabetes. Int Arch Occup Environ Health.

[23] Hansen AB, Stayner L, Hansen J (2016). Night shift work and incidence of diabetes in the Danish Nurse Cohort. Occup Environ Med.

[24] Andersson T, Alfredsson L, Källberg H (2005). Calculating measures of biological interaction. Eur J Epidemiol.

[25] Kawakami N, Araki S, Takatsuka N (1999). Overtime, psychosocial working conditions, and occurrence of non-insulin dependent type 2 diabetes in Japanese men. J Epidemiol Community Health.

[26] Suwazono Y, Sakata K, Okubo Y (2006). Long-term longitudinal study on the relationship between alternating shift work and the onset of type 2 diabetes in male Japanese workers. J Occup Environ Med.

[27] Pan A, Schernhammer ES, Sun Q (2011). Rotating night shift work and risk of type 2 diabetes: two prospective cohort studies in women. PLoS Med.

[28] Vimalananda VG, Palmer JR, Gerlovin H (2015). Night-shift work and incident diabetes among African-American women. Diabetologia.

[29] Axelsson J, Puttonen S (2012). Night shift work increases the risk for type 2 diabetes. Evid Based Med.

[30] Vetter C, Dashti HS, Lane JM (2018). Night shift work, genetic risk, and type 2 diabetes in the UK biobank. Diabetes Care.

[31] Lyssenko V, Nagorny CL, Erdos MR (2009). Common variant in MTNR1B associated with increased risk of type 2 diabetes and impaired early insulin secretion. Nat Genet.

[32] Bouatia-naji N, Bonnefond A, Cavalcanti-proença C (2009). A variant near MTNR1B is associated with increased fasting plasma glucose levels and type 2 diabetes risk. Nat Genet.

[33] Florez JC, JablonskI KA, Mcateer JB (2012). Effects of genetic variants previously associated with fasting glucose and insulin in the Diabetes Prevention Program. PLoS One.

[34] Xia Q, Chen ZX, Wang YC (2012). Association between the melatonin receptor 1B gene polymorphism on the risk of type 2 diabetes, impaired glucose regulation: a meta-analysis. PLoS One.

[35] Prokopenko I, Langenberg C, Florez JC (2009). Variants in MTNR1B influence fasting glucose levels. Nat Genet.

[36] Young MW, Kay SA (2001). Time zones: a comparative genetics of circadian clocks. Nat Rev Genet.

[37] Borgs L, Beukelaers P, Vandenbosch R (2009). Cell "circadian" cycle: new role for mammalian core clock genes. Cell Cycle.

[38] FerrelL JM, Chiang JY (2015). Circadian rhythms in liver metabolism and disease. Acta Pharm Sin B.

[39] Dibner C, Schibler U, Albrecht U (2010). The mammalian circadian timing system: organization and coordination of central and peripheral clocks. Annu Rev Physiol.

[40] Stamenkovic JA, Olsson AH, Nagorny CL (2012). Regulation of core clock genes in human islets. Metabolism.

[41] Lamia KA, Evans RM (2010). Metabolism: tick, tock, a beta-cell clock. Nature.

[42] Dubocovich ML, Markowska M (2005). Functional MT1 and MT2 melatonin receptors in mammals. Endocrine.

[43] Li C, Qiao B, Zhan Y (2013). Association between genetic variations in MTNR1A and MTNR1B genes and gestational type 2 diabetes in Han Chinese women. Gynecol Obstet Invest.

[44] Hardeland R, Madrid JA, Tan DX (2012). Melatonin, the circadian multioscillator system and health: the need for detailed analyses of peripheral melatonin signaling. J Pineal Res.

