# Supplementary material for: Association of rotating night shift work, CLOCK, MTNR1A, MTNR1B genes polymorphisms and their interactions with type 2 diabetes among steelworkers: a case–control study
Source: BMC Genomics. 2023 May 3;24:232. doi: 10.1186/s12864-023-09328-y (PMC10157991; doi:10.1186/s12864-023-09328-y)
Supplement: Supplementary file 1 — Additional file 1. [file 12864_2023_9328_MOESM1_ESM.pdf]

Association of rotating night shift work, CLOCK, MTNR1A, MTNR1B genes polymorphisms and their interactions with type 2 diabetes among steelworkers: A case-control study

Qinglin Li[a,#], Shengkui Zhang[a,#], Wang Han[d], Zhende Wang[c], Xiaohong Zhang[a], Yongbin Wang[b,*], Juxiang Yuan[a,*]

[a] Department of Epidemiology and Health Statistics, School of Public Health, North China University of Science and Technology, Tangshan, Hebei, China

[b] Department of Epidemiology and Health Statistics, School of Public Health, Xinxiang Medical University, Xinxiang, Henan Province, P.R. China

[c] Department of Public health crisis management, School of Public Health, Weifang Medical University, Weifang, Shandong Province, P.R. China

[d] Tianjin Baodi District Center for Disease Control

[#] These authors contributed equally to this work.

***Corresponding author:** Professor Juxiang Yuan, School of Public Health, North China University of Science and Technology, Tangshan, Hebei, China; Tel: +13603256766; E-mail: jxyuan5082@163.com and yuanjx@ncst.edu.cn; And professor Yongbin Wang, School of Public Health, Xinxiang Medical University, Xinxiang, Henan Province, P.R. China; E-mail: wybwho@163.com.

Assessment of covariates

Smoking status was divided into "never", "ever" and "current". Drinking status was evaluated from self-reported information, mainly including the amount and frequency of alcohol consumed per week and was divided into "never", "ever" and "current". Those who usually consumed some alcohol at least once a week over the past 12 months were defined as current drinkers. For current drinkers, the frequency of drinking status (days/week), usually the average amount of alcohol consumed (g), and types of beverages were recorded. Alcohol content across different types of beverages in China was assessed as follows: strong spirits 53%, weak spirits 38%, rice wine 15%, grape wine 12%, and beer 4%. Based on the beverage type, amount consumed, and frequency, we were able to derive the amount of pure alcohol (g/week) consumed per week.[1] Intake levels of two standard drinks (20 g ethanol daily, 140 g weekly) daily for males and one standard drink daily (70 g weekly) for females were considered acceptable thresholds for defining non-alcoholics.[2] In this study, we have eliminated individuals with excessive drinking status. Participants were asked how many hours of sleep each day when they go to different shifts (morning shift, afternoon shift, and night shift) and how many hours of sleep each day when they rest. Sleep duration was the weighted mean of sleep duration on working days and non-working days. In the four-crew-three-shift system, sleep duration = [(sleep duration of morning shift × 2) + (sleep duration of afternoon shift × 2) + (sleep duration of night shift × 2) + (sleep duration at rest × 2)]/8. In the three-crew-two-shift system, sleep duration = [(sleep duration of morning shift × 1) +

(sleep duration of night shift × 1) + (sleep duration at rest × 1)]/3. For workers who never work shifts, sleep duration was a weighted average of the working and nonworking days of a work/rest cycle.

Smoking and drinking status: never, ever, current. The calculation of metabolic equivalents was based on the International Physical Activity Questionnaire (IPAQ)[3]. Physical activity (MET- hours/week) was divided into "low", "middle" and "high". DASH score: Score according to (dietary approaches to stop hypertension, DASH)[4]. Height and weight were measured three times each. The participants stood upright and barefoot in light clothes. The height and weight data that were ultimately used for analysis were accurate to 0.1 cm and 0.1 kg. Body mass index (BMI) was defined as body weight (kg) divided by the square of the body height (m^2). Blood pressure measurements were performed three times five-minute intervals using an electronic sphygmomanometer (OMRON, HBP-1100, China), and the participants were required to rest for more than ten minutes. Finally, the mean was obtained for analysis. Elevated blood pressure was defined as current systolic blood pressure ≥140 mmHg, or diastolic blood pressure ≥90 mmHg, or if the patient was receiving antihypertensive therapy. Total cholesterol (TC) ≥6.22 mmol/L or low-density lipoprotein (LDL-C) ≥4.11 mmol/L or high-density lipoprotein (HDL-C) ≤1.04 mmol/L or triglycerides (TG) ≥2.32 mmol/L, or patients undergoing lipid-lowering therapy were considered to demonstrate dyslipidaemia. Renal function damage: estimated glomerular filtration rate (GFR) <90 ml/min/1.73 m^2 was defined as decreased eGFR[5].

Occupational hazards (high temperature, noise, dust and carbon monoxide (CO)) exposure: yes or no. Exposure to dust was defined as workers who may be exposed to productive dust (inorganic dust, organic dust or mixed dust) during production (GBZ/T 229.1−2010).[6] The total dust in the air of workplace was collected at the breathing zone with a filter membrane, and it's concentration was calculated based on the increased weight of the filter membrane and the amount of gas collected. When the dust concentration in the air ≤50mg/m3, a filter membrane with a diameter of 37mm or 40mm was used, otherwise a filter membrane with a diameter of 75mm would be used (GBZ/T192.1–2007).[7] Exposure to high temperature (heat stress work) was defined as the average wet-bulb globe temperature (WBGT) index of the workplace is equal or greater than 25 ℃ in the process of production (GBZ 2.2−2007).[8] The WBGT index was measured by black-wet bulb globe thermometer. If there was no productive heat source in the workplace, three measuring points were selected to take the average value of WBGT index, while where there was a productive heat source, 3 to 5 measuring points were selected to take the average value of WBGT index. If the workplace was isolated into different thermal or ventilated environment, 2 measuring points were selected to take the average value of WBGT index (GBZ/T 189.7–2007).[9] Exposure to industrial toxicant was defined as workers who may be exposed to a variety of harmful chemicals (the toxicant specifically refers to carbon monoxide in this population) during production (GBZ/T 229.2−2010).[10] Carbon monoxide or carbon dioxide in the air of workplace was pumped into the Non-Dispersive Infrared- Ray (NDIR) analyzer and selectively

absorbs their infrared rays. The concentration of carbon monoxide was determined according to the absorption value (GBZ/T 160.28－2004).[11] Exposure to noise was defined as workers who exposed to a noisy environment where the 8h/d or 40h/week equivalent A-weighted sound pressure level is ≥80dB, which may be harmful to health and hearing (GBZ/T 229.4－2012).[12] The workplace production noise was measured by a sound level meter. If the distribution of sound field in the workplace was uniform (between-field difference of A-sound levels were less than 3dB(A)), three measuring points were selected to take the average value, otherwise it should be divided into several sound level areas. In each sound field, two measuring points were selected to take the average value (GBZ/T 189.8–2007).[13]

Table of contents

Table S1 The primer sequence and Tm value for target SNPs

Table S2 The PCR reaction system (20μl)

Table S3 The reaction condition for target SNP

Table S4 The enzyme digestion reaction system and conditions for target SNPs

Table S5 The relationship between MTNR1A gene rs2119882 locus, CLOCK gene rs1801260 locus and type 2 diabetes among steelworkers

Table S6 Multiplicative interactions between current shift status and genes on the risk of type 2 diabetes

Table S7 Multiplicative interactions between the duration of night shifts and genes on the risk of type 2 diabetes

Table S8 Multiplicative interactions between the average frequency of night shifts

and genes on the risk of type 2 diabetes

Table S9 Multiplicative and additive gene -gene interactions on the risk of type 2 diabetes

Table S10 The association between gene and type 2 diabetes among steelworkers stratified by rs2119882 and rs1801260

Table S11 The association between gene, rotating night shift work and type 2 diabetes among steelworkers ratified by rs1387153 and rs2119882

Table S12 The statistical test results for the best-performing interaction models based on the GMDR

Table S13 Odds ratios for type 2 diabetes according to different exposure metrics of night shift work stratified by current shift status

Table S1 The primer sequence and Tm value for target SNPs

Gene	Locus		Primer sequence	Tm (°C)
CLOCK	rs1801260	F	5'-TCCAGCAGTTTCATGAGATGC-3'	55.2
		R	5'-GAGGTCATTTCATAGCTGAGC-3'	53.1
MTNR1A	rs2119882	F	5-CCGTTCATTGTGTTTCCT-3	49.6
		R	5'-AGACAGTCCTTGGTTTTTC-3'	49.7
MTNR1B	rs1387153	F	5'-CCAAGGACACCCAGTTAGTGAG-3'	57.7
		R	5'-CCACCTTTATGCAGAACACCTTAT-3'	54.8

Table S2 The PCR reaction system (20μ l)

Composition	volume（μl）
PCR Taq Master Mix	10
Forward primer	1
Reverse primer	1
DNA template	2
Deionized water	6

Table S3 The reaction condition for target SNP

Gene	Locus	Pre–denaturation	Denaturation	Annealing	Extension	Number of cycles	Terminal extension
CLOCK	rs1801260	94℃3min	94℃40s	54℃30s	72℃60s	35	72℃5min
MTNR1A	rs2119882	94℃5min	96℃30s	52℃45s	72℃30s	35	72℃5min
MTNR1B	rs1387153	95℃7min	94℃30s	57℃50s	72℃60s	35	72℃10min

Table S4 The enzyme digestion reaction system and conditions for target SNPs

Gene	Locus	PCR products (μl）	Endonuclease (U)	Restriction sites	pure water (μl）	Reaction conditions
CLOCK	rs1801260	5	Bsp1286I	5'…GDGCH^C…3'	1	37 ℃ water bath for 16h
MTNR1A	rs2119882	5	AvaII	5'…G^GWCC…3'	1	37℃ water bath for 15min
MTNR1B	rs1387153	5	MvaI	5'…CC^WGG…3'	1	60℃ water bath for 3h

Table S5 The relationship between MTNR1A gene rs2119882 locus, CLOCK gene rs1801260 locus and type 2 diabetes among steelworkers

Gene	Gene Model	Genotype	Control group, n (%)	Case group, n(%)	Model 1	Model 2
					OR (95% CI)	OR (95% CI)
MTNR1A gene rs2119882 locus	Codominant	T/T	153 (33.9)	98 (39.0)	1.00 (ref)	1.00 (ref)
		C/T	217 (48.1)	120 (47.8)	0.86 (0.62–1.21)	0.88 (0.62–1.26)
		C/C	81 (18.0)	33 (13.2)	0.64 (0.39–1.03)	0.67 (0.41–1.10)
	Dominant	T/T	153 (33.9)	98 (39.0)	1.00 (ref)	1.00 (ref)
		C/T-C/C	298 (66.1)	153 (61.0)	0.80 (0.58–1.10)	0.83 (0.59–1.15)
	Recessive	T/T-C/T	370 (82.0)	218 (86.8)	1.00 (ref)	1.00 (ref)
		C/C	81 (18.0)	33 (13.2)	0.69 (0.45–1.07)	0.72 (0.45–1.13)
	Overdominant	T/T-C/C	234 (51.9)	131 (52.2)	1.00 (ref)	1.00 (ref)
		C/T	217 (48.1)	120 (47.8)	0.99 (0.73–1.35)	0.99 (0.72–1.37)
	Log-additive	---	---	---	0.81 (0.65–1.02)	0.83 (0.66–1.05)
CLOCK gene rs1801260 locus	Codominant	T/T	387 (85.8)	204 (81.3)	1.00 (ref)	1.00 (ref)
		C/T	59 (13.1)	44 (17.5)	1.41 (0.92–2.17)	1.39 (0.89–2.18)
		C/C	5 (1.1)	3 (1.2)	1.14 (0.27–4.81)	1.25 (0.28–5.52)
	Dominant	T/T	387 (85.8)	204 (81.3)	1.00 (ref)	1.00 (ref)
		C/T-C/C	64 (14.2)	47 (18.7)	1.39 (0.92–2.11)	1.38 (0.89–2.13)
	Recessive	T/T-C/T	446 (98.9)	248 (98.8)	1.00 (ref)	1.00 (ref)
		C/C	5 (1.1)	3 (1.2)	1.08 (0.26–4.55)	1.20 (0.27–5.32)
	Overdominant	T/T-C/C	392 (86.9)	207 (82.5)	1.00 (ref)	1.00 (ref)
		C/T	59 (13.1)	44 (17.5)	1.41 (0.92–2.16)	1.39 (0.88–2.18)
	Log-additive	---	---	---	1.32 (0.91–1.91)	1.31 (0.89–1.94)

Model 1: univariate analysis. Model 2: adjusted for age, sex, smoking status, drinking status, physical activity, DASH score, dyslipidaemia, hypertension, liver dysfunction, renal dysfunction and exposure to occupational hazards (high temperature, noise, dust and carbon monoxide (CO)) in each exposure metric. DASH, dietary approaches to stop hypertension;

Table S6 Multiplicative interactions between current shift status and genes on the risk of type 2 diabetes

Genotype	Factors	Control group, n	Case group, n	*OR* (95% CI)		*P* for multiplicative interaction	
				Model 1	Model 2	Model 1	Model 2
rs2119882	Current shift status					0.326	0.254
T/T	Never	38	12	1.00 (ref)	1.00 (ref)		
	Ever	45	25	1.76 (0.78–3.96)	1.76 (0.76–4.07)		
	Current	70	61	**2.76 (1.32–5.75)**	**2.75 (1.29–5.87)**		
C/T-C/C	Never	67	20	1.00 (ref)	1.00 (ref)		
	Ever	79	44	1.87 (1.00–3.47)	1.90 (1.00–3.61)		
	Current	152	89	**1.96 (1.12–3.45)**	**1.83 (1.02–3.29)**		
rs1387153	Current shift status					0.015	0.012
C/C	Never	27	11	1.00 (ref)	1.00 (ref)		
	Ever	44	24	1.34 (0.57–3.16)	1.30 (0.54–3.14)		
	Current	86	37	1.06 (0.47–2.35)	0.91 (0.40–2.09)		
C/T-T/T	Never	78	21	1.00 (ref)	1.00 (ref)		
	Ever	80	45	**2.09 (1.14–3.82)**	**2.16 (1.15–4.03)**		
	Current	136	113	**3.09 (1.79–5.31)**	**3.07 (1.74–5.40)**		
rs1801260	Current shift status					0.576	0.652
T/T	Never	92	26	1.00 (ref)	1.00 (ref)		
	Ever	104	60	**2.04 (1.19–3.50)**	**2.12 (1.22–3.69)**		
	Current	191	118	**2.19 (1.34–3.58)**	**2.15 (1.29–3.59)**		
C/T-C/C	Never	13	6	1.00 (ref)	1.00 (ref)		
	Ever	20	9	0.98 (0.28–3.39)	0.84 (0.23–3.07)		
	Current	31	32	2.24 (0.75–6.63)	1.84 (0.60–5.67)		

Model 1: univariate analysis. Model 2: adjusted for age, sex, smoking status, drinking status, physical activity, DASH score, dyslipidaemia, hypertension, liver dysfunction, renal dysfunction and exposure to occupational hazards (high temperature, noise, dust and carbon monoxide (CO)) in each exposure metric. DASH, dietary approaches to stop hypertension; Value in bold: it indicates that it is statistically significant.

Table S7 Multiplicative interactions between the duration of night shifts and genes on the risk of type 2 diabetes

Genotype	Factors	Control group, n	Case group, n	*OR* (95% CI)		*P* for multiplicative interaction	
				Model 1	Model 2	Model 1	Model 2
rs2119882	Duration of night shifts (years)					0.114	0.071
T/T	Never	38	12	1.00 (ref)	1.00 (ref)		
	1-10	43	19	1.40 (0.60–3.25)	1.48 (0.62–3.56)		
	10-20	21	17	**2.56 (1.03–6.38)**	**3.23 (1.24–8.40)**		
	20-30	39	35	**2.84 (1.29–6.28)**	**2.75 (1.21–6.22)**		
	>30	12	15	**3.96 (1.46–10.75)**	2.76 (0.97–7.80)		
C/T-C/C	Never	67	20	1.00 (ref)	1.00 (ref)		
	1-10	56	30	1.79 (0.92–3.50)	**2.08 (1.03–4.21)**		
	10-20	56	30	1.79 (0.92–3.50)	**2.08 (1.03–4.20)**		
	20-30	79	52	**2.21 (1.20–4.06)**	**2.01 (1.07–3.78)**		
	>30	40	21	1.76 (0.85–3.64)	1.14 (0.53–2.47)		
rs1387153	Duration of night shifts (years)					0.016	0.009
C/C	Never	27	11	1.00 (ref)	1.00 (ref)		
	1-10	34	17	1.23 (0.49–3.05)	1.30 (0.50–3.36)		
	10-20	36	16	1.09 (0.44–2.73)	1.13 (0.44–2.93)		
	20-30	37	20	1.33 (0.55–3.22)	1.16 (0.47–2.87)		
	>30	23	8	0.85 (0.29–2.48)	0.48 (0.15–1.50)		
C/T-T/T	Never	78	21	1.00 (ref)	1.00 (ref)		
	1-10	65	32	1.83 (0.96–3.47)	**2.06 (1.05–4.05)**		
	10-20	41	31	**2.81 (1.44–5.49)**	**3.62 (1.78–7.38)**		
	20-30	81	67	**3.07 (1.72–5.49)**	**2.94 (1.61–5.39)**		
	>30	29	28	**3.59 (1.77–7.28)**	**2.52 (1.20–5.31)**		
rs1801260	Duration of night shifts (years)					0.776	0.828
T/T	Never	92	26	1.00 (ref)	1.00 (ref)		
	1-10	87	39	1.59 (0.89–2.82)	1.82 (0.99–3.33)		
	10-20	67	41	**2.17 (1.21–3.88)**	**2.67 (1.44–4.95)**		
	20-30	95	71	**2.64 (1.55–4.51)**	**2.47 (1.42–4.30)**		
	>30	46	27	**2.08 (1.09–3.96)**	1.44 (0.73–2.85)		
C/T-C/C	Never	13	6	1.00 (ref)	1.00 (ref)		

1-10	12	10	1.81 (0.50–6.50)	1.67 (0.43–6.52)
10-20	10	6	1.30 (0.32–5.27)	1.21 (0.28–5.23)
20-30	23	16	1.51 (0.47–4.80)	1.34 (0.41–4.43)
>30	6	9	3.25 (0.79–13.38)	1.82 (0.42–7.90)

Model 1: univariate analysis. Model 2: adjusted for age, sex, smoking status, drinking status, physical activity, DASH score, dyslipidaemia, hypertension, liver dysfunction, renal dysfunction and exposure to occupational hazards (high temperature, noise, dust and carbon monoxide (CO)) in each exposure metric. DASH, dietary approaches to stop hypertension; Value in bold: it indicates that it is statistically significant.

Table S8 Multiplicative interactions between the average frequency of night shifts and genes on the risk of type 2 diabetes

Genotype	Factors	Control group, n	Case group, n	*OR* (95% CI)		*P* for multiplicative interaction	
				Model 1	Model 2	Model 1	Model 2
rs2119882	Average frequency of night shifts (nights/month)					0.811	0.853
T/T	Never	38	12	1.00 (ref)	1.00 (ref)		
	<3	35	25	2.26 (0.99–5.17)	2.24 (0.95–5.30)		
	3-8	73	52	**2.26 (1.08–4.73)**	**2.20 (1.03–4.71)**		
	>8	7	9	**4.07 (1.25–13.28)**	**4.50 (1.31–15.46)**		
C/T-C/C	Never	67	20	1.00 (ref)	1.00 (ref)		
	<3	80	37	1.55 (0.82–2.92)	1.36 (0.70–2.64)		
	3-8	133	82	**2.07 (1.17–3.65)**	**2.07 (1.15–3.74)**		
	>8	18	14	**2.61 (1.10–6.15)**	2.35 (0.96–5.78)		
rs1387153	Average frequency of night shifts (nights/month)					0.459	0.415
C/C	Never	27	11	1.00 (ref)	1.00 (ref)		
	<3	44	15	0.84 (0.34–2.09)	0.65 (0.25–1.70)		
	3-8	79	39	1.21 (0.54–2.69)	1.17 (0.51–2.66)		
	>8	7	7	2.45 (0.70–8.66)	2.02 (0.54–7.55)		
C/T-T/T	Never	78	21	1.00 (ref)	1.00 (ref)		
	<3	71	47	**2.46 (1.34–4.51)**	**2.39 (1.27–4.51)**		
	3-8	127	95	**2.78 (1.60–4.82)**	**2.77 (1.57–4.91)**		
	>8	18	16	**3.30 (1.44–7.56)**	**3.49 (1.47–8.29)**		

rs1801260	Average frequency of night shifts (nights/month)					0.644	0.651
T/T	Never	92	26	1.00 (ref)	1.00 (ref)		
	<3	100	56	**1.98 (1.15–3.42)**	**1.87 (1.06–3.30)**		
	3-8	175	106	**2.14 (1.30–3.53)**	**2.22 (1.32–3.71)**		
	>8	20	1	**2.83 (1.29–6.23)**	**2.72 (1.19–6.23)**		
C/T-C/C	Never	13	6	1.00 (ref)	1.00 (ref)		
	<3	15	6	0.87 (0.22–3.36)	0.66 (0.16–2.81)		
	3-8	31	28	1.96 (0.66–5.84)	1.59 (0.51–4.95)		
	>8	5	7	3.03 (0.68–13.61)	2.89 (0.61–13.67)		

Model 1: univariate analysis. Model 2: adjusted for age, sex, smoking status, drinking status, physical activity, DASH score, dyslipidaemia, hypertension, liver dysfunction, renal dysfunction and exposure to occupational hazards (high temperature, noise, dust and carbon monoxide (CO)) in each exposure metric. DASH, dietary approaches to stop hypertension; Value in bold: it indicates that it is statistically significant.

Table S9 Multiplicative and additive gene-gene interactions on the risk of type 2 diabetes

Genotype1	Genotype2	Control group, n	Case group, n	OR (95% CI)		*P* for multiplicative interaction	
				Model 1	Model 2	Model 1	Model 2
rs1387153	rs2119882					0.964	0.989
C/C	T/T	54	26	1.00 (ref)	1.00 (ref)		
C/C	C/T-C/C	103	46	0.93 (0.52–1.66)	0.82 (0.45–1.50)		
C/T-T/T	T/T	99	72	1.51 (0.87–2.64)	1.32 (0.74–2.36)		
C/T-T/T	C/T-C/C	195	107	1.14 (0.68–1.92)	1.09 (0.64–1.88)		
	RERI			-0.30 (-0.16–0.57)	-0.05 (-0.82–0.72)		
	AP			-0.26 (-0.96–0.44)	-0.05 (-0.74–0.65)		
rs2119882	rs1801260					0.040	0.013
T/T	T/T	189	21	1.00 (ref)	1.00 (ref)		
T/T	C/T-C/C	34	7	0.78 (0.39–1.57)	0.65 (0.32–1.34)		
C/T-C/C	T/T	342	39	0.69 (0.49–0.98)	0.69 (0.47–1.00)		
C/T-C/C	C/T-C/C	57	13	1.34 (0.78–2.31)	1.39 (0.79–2.48)		
	RERI			**0.87 (0.05–1.69)**	**1.07 (0.23–1.91)**		
	AP			**0.65 (0.18–1.12)**	**0.77 (0.36–1.17)**		
rs1387153	rs1801260					0.457	0.616
C/C	T/T	126	84	1.00 (ref)	1.00 (ref)		
C/C	C/T-C/C	261	120	1.75 (0.86–3.59)	1.58 (0.74–3.38)		
C/T-T/T	T/T	27	14	1.42 (0.98–2.05)	1.39 (0.95–2.05)		
C/T-T/T	C/T-C/C	37	33	**1.78 (1.02–3.11)**	1.73 (0.97–3.09)		
	RERI			-0.39 (-1.88–1.10)	-0.24 (-1.69–1.22)		
	AP			-0.22 (-1.10–0.66)	-0.14 (-1.01–0.73)		

Model 1: univariate analysis. Model 2: adjusted for age, sex, smoking status, drinking status, physical activity, DASH score, dyslipidaemia, hypertension, liver dysfunction, renal dysfunction and exposure to occupational hazards (high temperature, noise, dust and carbon monoxide (CO)) in each exposure metric. DASH, dietary approaches to stop hypertension RERI: Relative excess risk due to interaction; AP: Attributable proportion. Value in bold: it indicates that it is statistically significant.

Table S10 The association between gene and type 2 diabetes among steelworkers stratified by rs2119882 and rs1801260

Genotype1	Genotype2	Control group, n	Case group, n	OR (95% CI)	
				Model 1	Model 2
rs2119882	rs1801260				
T/T	T/T	189	21	1.00 (ref)	1.00 (ref)
	C/T-C/C	34	7	0.78 (0.39–1.57)	0.65 (0.32–1.34)
C/T-C/C	T/T	342	39	1.00 (ref)	1.00 (ref)
	C/T-C/C	57	13	**1.94 (1.16–3.25)**	**1.87 (1.07–3.28)**
rs1801260	rs2119882				
T/T	T/T	189	21	1.00 (ref)	1.00 (ref)
	C/T-C/C	342	39	0.69 (0.49–0.98)	0.69 (0.47–1.00)
C/T-C/C	T/T	34	7	1.00 (ref)	1.00 (ref)
	C/T-C/C	57	13	**2.43 (1.03–5.76)**	**3.89 (1.22–12.42)**

Model 1: univariate analysis. Model 2: adjusted for age, sex, smoking status, drinking status, physical activity, DASH score, dyslipidaemia, hypertension, liver dysfunction, renal dysfunction and exposure to occupational hazards (high temperature, noise, dust and carbon monoxide (CO)) in each exposure metric. DASH, dietary approaches to stop hypertension RERI: Relative excess risk due to interaction; AP: Attributable proportion. Value in bold: it indicates that it is statistically significant.

Table S11 The association between gene, rotating night shift work and type 2 diabetes among steelworkers stratified by rs1387153 and rs2119882

Factor1	Factor2	Control group, n	Case group, n	OR (95% CI)	
				Model 1	Model 2
rs1387153	Rotating night shift work				
C/C	No	27	11	1.00 (ref)	1.00 (ref)
	Yes	78	21	1.15 (0.54–2.47)	0.99 (0.43–2.28)
C/T-T/T	No	130	61	1.00 (ref)	1.00 (ref)
	Yes	216	158	**2.72 (1.61–4.49)**	**2.66 (1.54–4.59)**
rs2119882	rs1801260				
T/T	T/T	189	21	1.00 (ref)	1.00 (ref)
	C/T-C/C	34	7	0.78 (0.39–1.57)	0.59 (0.27–1.27)
C/T-C/C	T/T	342	39	1.00 (ref)	1.00 (ref)
	C/T-C/C	57	13	**1.94 (1.16–3.25)**	**1.95 (1.12–3.40)**

Model 1: univariate analysis. Model 2: adjusted for age, sex, smoking status, drinking status, physical activity, DASH score, dyslipidaemia, hypertension, liver dysfunction, renal dysfunction and exposure to occupational hazards (high temperature, noise, dust and carbon monoxide (CO)) in each exposure metric. DASH, dietary approaches to stop hypertension; BMI: body mass index. Value in bold: it indicates that it is statistically significant.

Table S12 The statistical test results for the best-performing interaction models based on the GMDR

Dataset	χ^2	*P*	OR (95% CI)
Training	15.659	<0.001	2.64 (1.62–4.29)
Testing	0.926	0.336	1.87 (0.43–8.12)
Whole dataset	16.162	<0.001	2.49 (1.59–3.90)

Adjusted for age, sex, smoking status, drinking status, physical activity, DASH score, dyslipidaemia, hypertension, liver dysfunction, renal dysfunction and exposure to occupational hazards (high temperature, noise, dust and carbon monoxide (CO)). DASH, dietary approaches to stop hypertension; BMI: body mass index.

Table S13 Odds ratios for type 2 diabetes according to different exposure metrics of night shift work stratified by current shift status

Current shift status	Exposure metrics	Model 1	Model 2
		OR (95% *CI*)	*OR* (95% *CI*)
Ever/Never	Duration of night shifts (years)		
	Never	1.00 (ref)	1.00 (ref)
	1-10	1.25 (0.68–2.31)	1.55 (0.81–2.97)
	10-20	1.99 (0.97–1.10)	**2.35 (1.10–5.04)**
	20-30	**2.84 (1.47–5.49)**	**2.48 (1.24–4.97)**
	>30	2.19 (0.35–13.67)	0.83 (0.08–8.76)
	P trend	0.001	0.009
	Average frequency of night shifts (nights/month)		
	Never	1.00 (ref)	1.00 (ref)
	<3	1.54 (0.82–2.90)	1.61 (0.82–3.15)
	3-8	**2.09 (1.20–3.63)**	**2.30 (1.28–4.14)**
	>8	1.46 (0.42–5.05)	1.58 (0.43–5.77)
	P trend	0.018	0.011
Curent/Never	Duration of night shifts (years)		
	Never	1.00 (ref)	1.00 (ref)
	1-10	**2.28 (1.20–4.35)**	**2.61 (1.27–5.35)**
	10-20	**2.01 (1.10–3.67)**	**2.59 (1.34–5.03)**
	20-30	**2.28 (1.36–3.80)**	**2.03 (1.18–3.49)**
	>30	**2.28 (1.26–4.11)**	1.39 (0.73–2.64)
	P trend	0.003	0.131
	Average frequency of night shifts (nights/month)		
	Never	1.00 (ref)	1.00 (ref)
	<3	**1.94 (1.11–3.39)**	1.63 (0.90–2.97)
	3-8	**2.16 (1.34–3.47)**	**2.05 (1.25–3.37)**
	>8	**3.90 (1.80–8.45)**	**3.13 (1.37–7.14)**
	P trend	<0.001	0.001

Model 1: univariate analysis. Model 2: adjusted for age, sex, smoking status, drinking status, physical activity, DASH score, dyslipidaemia, hypertension, liver dysfunction, renal dysfunction and exposure to occupational hazards (high temperature, noise, dust and carbon monoxide (CO)). DASH, dietary approaches to stop hypertension; BMI: body mass index. Value in bold: it indicates that it is statistically significant.

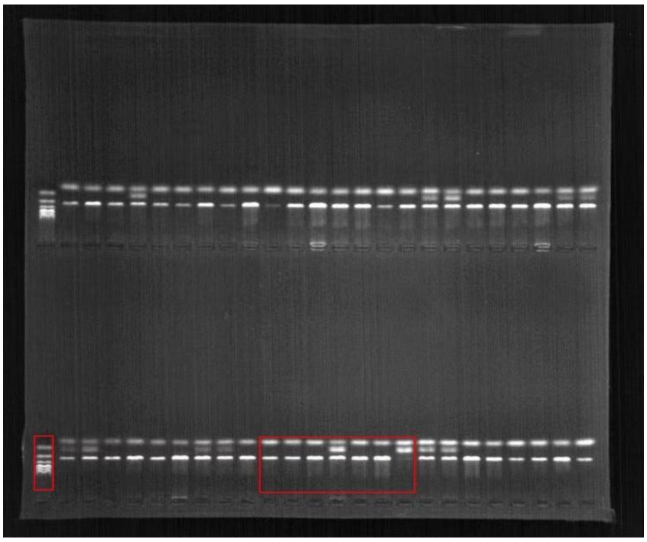

Figure S1 The original electrophoretic gel results of rs1801260 locus for CLOCK gene

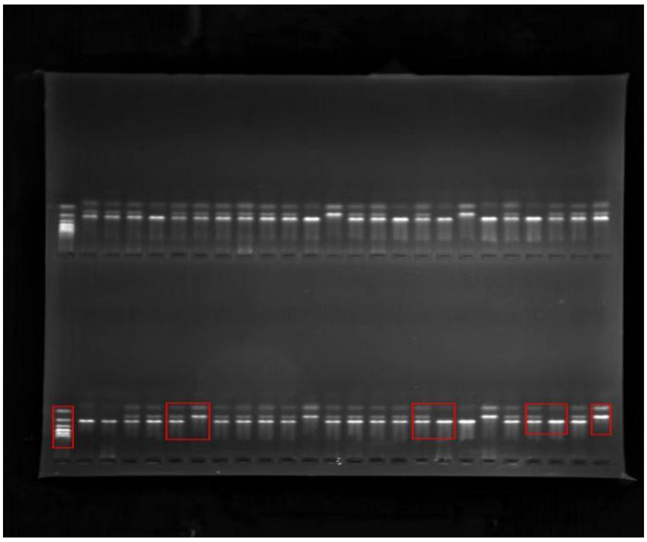

Figure S2 The original electrophoretic gel results of rs2119882 locus for MTNR1A gene

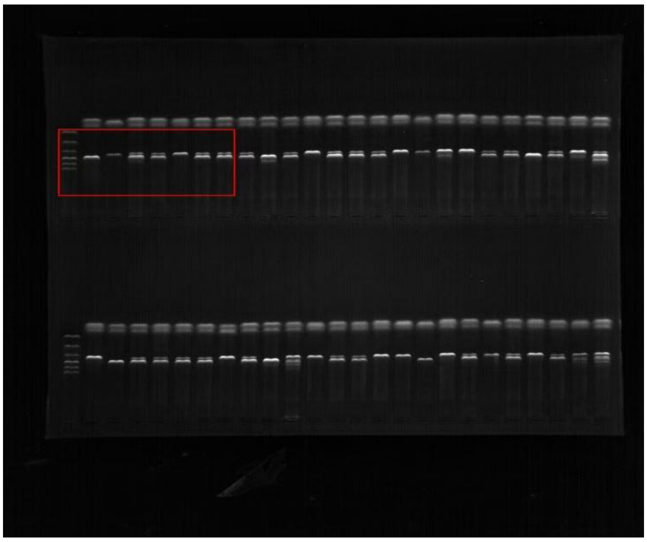

Figure S3 The original electrophoretic gel results of rs1387153 locus for MTNR1B gene

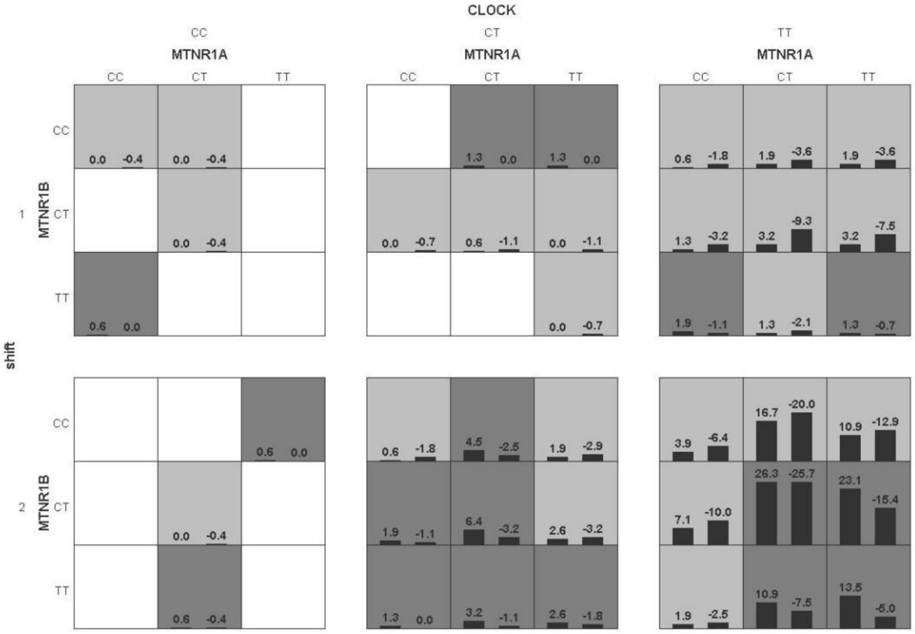

Figure S4 The combined model of genes–rotating night shift work interactions.

Adjusted for age, sex, smoking status, drinking status, sleep duration, BMI, physical activity, DASH score, dyslipidaemia, hypertension, liver dysfunction, renal dysfunction and exposure to occupational hazards (high temperature, noise, dust and carbon monoxide (CO)). DASH, dietary approaches to stop hypertension; BMI: body mass index; shift: rotating night shift work.

REFERENCES

[1] Millwood IY, Walters RG, Mei XW, et al; China Kadoorie Biobank Collaborative Group. Conventional and genetic evidence on alcohol and vascular disease aetiology: a prospective study of 500 000 men and women in China. Lancet. 2019 May 4;393(10183):1831-1842. doi: 10.1016/S0140-6736(18)31772–0.

[2] Farrell GC, Chitturi S, Lau GK, et al; Asia-Pacific Working Party on NAFLD. Guidelines for the assessment and management of non-alcoholic fatty liver disease in the Asia-Pacific region: executive summary. J Gastroenterol Hepatol. 2007 Jun;22(6):775–7. doi: 10.1111/j.1440–1746.2007.05002.x.

[3] Celis–Morales CA, Perez–Bravo F, Ibanez L, et al. Objective vs. self–reported physical activity and sedentary time: effects of measurement method on relationships with risk biomarkers. PLoS One. 2012;7(5):e36345. doi: 10.1371/journal.pone.0036345.

[4] Maskarinec g, lim U, Jacobs s, et al. Diet quality in Midadulthood predicts visceral adiposity and liver fatness in older ages: the Multiethnic cohort study. Obesity. 2017;25:1442–50. doi: 10.1002/oby.21868. Erratum in: Obesity (Silver Spring). 2018 Jan;26(1):239.

[5] Webster AC, Nagler EV, Morton RL, Masson P. Chronic Kidney Disease. Lancet. 2017 Mar 25;389(10075):1238–1252. doi: 10.1016/S0140–6736(16)32064–5.

[6] GBZ/T 229.1–2010 GBZ/T 229.1–2010 Classification of occupational hazards at workplaces. Part 1: Occupational exposure to industrial dust. http://niohp.chinacdc.cn/zyysjk/zywsbzml/201210/t20121012_70490.htm.

Accessed 12 January 2021.

[7] GBZ/T 192.1–2007 Determination of dust in the air of workplace. Part 1: Total dust concentration. http://niohp.chinacdc.cn/zyysjk/zywsbzml/201210/t20121012_70522.htm. Accessed 12 January 2021.

[8] GBZ 2.2–2007 Occupational exposure limits for hazardous agents in the workplace. Part 2: Physical agents. http://niohp.chinacdc.cn/zyysjk/zywsbzml/201303/t20130329_79199.htm. Accessed 12 January 2021.

[9] GBZ/T 189.7–2007 Measurement of physical agents in workplace. Part 7: Heat Stress. http://niohp.chinacdc.cn/zyysjk/zywsbzml/201210/t20121012_70527.htm. Accessed 12 January 2021.

[10] GBZ/T 229.2–2010 Classification of occupational hazards at workplaces. Part 2: Occupational exposure to chemicals. http://niohp.chinacdc.cn/zyysjk/zywsbzml/201210/t20121012_70489.htm. Accessed 12 January 2021.

[11] GBZ/T 160.28–2004 Methods for determination of inorganic carbon compounds in the air of workplace. http://niohp.chinacdc.cn/zyysjk/zywsbzml/201210/t20121015_70624.htm. Accessed 12 January 2021.

[12] GBZ/T 229.4–2012 Classification of occupational hazards at workplaces. Part 4: Occupational exposure to noise.

http://niohp.chinacdc.cn/zyysjk/zywsbzml/201307/t20130715_84934.htm. Accessed 12 January 2021.

[13] GBZ/T 189.8–2007 Measurement of physical agents in workplace. Part 8: Noise. http://niohp.chinacdc.cn/zyysjk/zywsbzml/201210/t20121012_70526.htm. Accessed 12 January 2021.
